# Interactive effects of music and prefrontal cortex stimulation in modulating response inhibition

**DOI:** 10.1038/s41598-017-18119-x

**Published:** 2017-12-22

**Authors:** Farshad Alizadeh Mansouri, Nicola Acevedo, Rosin Illipparampil, Daniel J. Fehring, Paul B. Fitzgerald, Shapour Jaberzadeh

**Affiliations:** 1 Department of Physiology, Cognitive Neuroscience Laboratory, Monash Biomedicine Discovery Institute, Monash University, Victoria, 3800 Australia; 2 Department of Physiotherapy, Non-invasive Brain Stimulation & Neuroplasticity Laboratory, Monash University, Victoria, 3199 Australia; 3 ARC Centre of Excellence in Integrative Brain Function, Monash University, Victoria, Australia; 4 Monash Alfred Psychiatry Research Centre, Central Clinical School, Monash University and the Alfred Hospital, Victoria, Australia

## Abstract

Influential hypotheses propose that alterations in emotional state influence decision processes and executive control of behavior. Both music and transcranial direct current stimulation (tDCS) of prefrontal cortex affect emotional state, however interactive effects of music and tDCS on executive functions remain unknown. Learning to inhibit inappropriate responses is an important aspect of executive control which is guided by assessing the decision outcomes such as errors. We found that high-tempo music, but not low-tempo music or low-level noise, significantly influenced learning and implementation of inhibitory control. In addition, a brief period of tDCS over prefrontal cortex specifically interacted with high-tempo music and altered its effects on executive functions. Measuring event-related autonomic and arousal response of participants indicated that exposure to task demands and practice led to a decline in arousal response to the decision outcome and high-tempo music enhanced such practice-related processes. However, tDCS specifically moderated the high-tempo music effect on the arousal response to errors and concomitantly restored learning and improvement in executive functions. Here, we show that tDCS and music interactively influence the learning and implementation of inhibitory control. Our findings indicate that alterations in the arousal-emotional response to the decision outcome might underlie these interactive effects.

## Introduction

Inhibition of inappropriate responses is an important aspect of executive control of behavior^1^ and patients with neuropsychiatric disorders show deficits in such executive functions^2–4^. Music is a commonly encountered contextual factor that affects arousal and emotional state^5–12^. Music has gained attention for potential use in regulating mood^13^, and managing learning disabilities and as an adjunct in rehabilitation of neuropsychiatric disorders^7,14–18^. Previous studies have shown that background music might exert no effect^19,20^, positive influence^7,9–12,21–25^ or even negative effect^20,25–28^ on cognitive functions. The presence of a goal-irrelevant background stimulus, such as music, might decrease attentional resources and increase distractibility^28–31^. The negative effects of music might be related to the engagement of cognitive resources and consequently in limiting the allocation of adequate amount of resources to the ongoing task. Listening to music activates prefrontal cortical areas that are involved in supporting executive functions^32–35^ and therefore music might directly exert negative or positive influence on cognitive processes. The effects of music might also be mediated through alterations in emotional state that affect focusing on the currently performed task^7,9,36^. Different features of music may affect the impact of music on arousal/emotional state^19,36–39^. Among these music features, tempo is better quantifiable and less subjective and previous studies indicate that high-tempo music has a small but significant effect in increasing the speed of motor behavior^28^ and is linked with arousal level^37^ while low-tempo music adversely affects reading efficiency^38^.

Practice-related learning processes induce plastic alterations in dorsolateral prefrontal cortex (DLPFC) and anterior cingulate cortex (ACC)^39,40^ and can potentially improve inhibition ability to manage compulsive behavior such as addiction^41^. Recent studies suggest that transcranial direct current stimulation (tDCS) of DLPFC might improve mnemonic and executive functions and may be used for managing neuropsychological disorders^42–45^. An influential hypothesis proposes that alterations in emotional and arousal state during cognitive task performance influence the decision process^46,47^. Both music and tDCS^42–44,48^ might independently alter mood and emotional state and influence cognitive processes, however it is still unclear whether and how tDCS and music interact in altering arousal-emotional state and cognitive functions. tDCS might alter the neural network state and change its susceptibility to the music effects^32–34,42–44^. We hypothesized that music and tDCS might modulate arousal/emotional state, which are associated with emotional and cognitive control and consequently influence learning and implementation of executive functions in the context of goal-directed behaviours.

**Figure 1:**
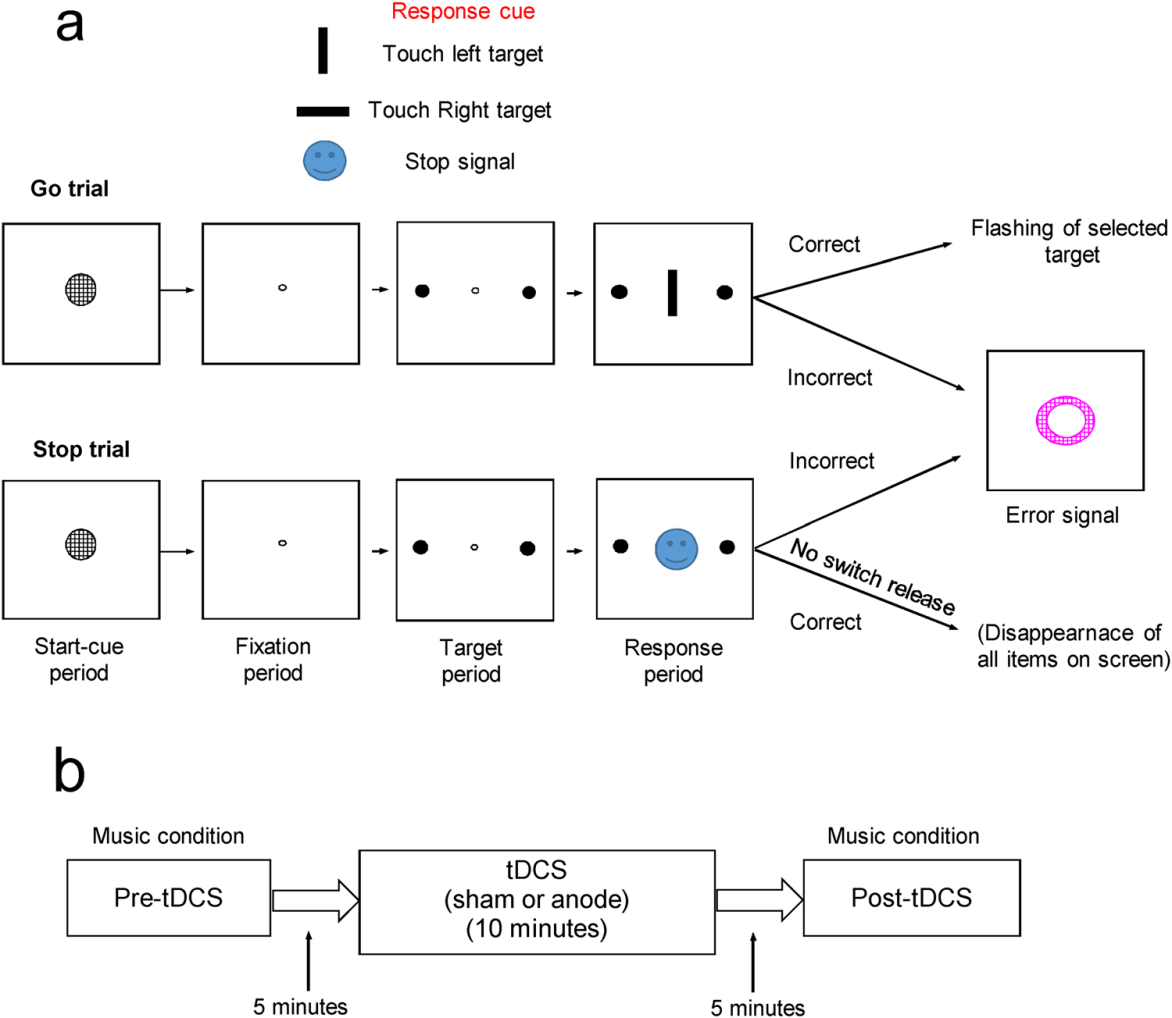
The Stop signal task and tDCS procedure. (**a**) In Go trials, first a Start-cue (grey circle) appeared and instructed pushing a switch (located at the bottom of the monitor) with the right hand within 10 seconds. Electrodermal activity was recorded from the left hand. The switch pressing changed the start cue to a fixation point. Participants kept the switch pressed during fixation period (450 ms). Then, two small targets (white circles) appeared for 300 ms at the left and right side of the fixation point. If the subject maintained switch pressing the fixation point was turned off and a go cue (horizontal or vertical white bar) was presented (black background). Horizontal bar instructed touching the right-side target (right Go trial) but vertical bar instructed touching the left-side target (left Go trial). Within a limited time window (900 ms from the onset of the go cue) participants had to release the switch and touch the target. Failure to touch the screen in this time window was considered as time-out errors. After correct target selection a feedback was given to the subjects (the selected target flashed twice). After an erroneous target selection or early release of the switch, all the items were turned off and a visual error signal (a purple annulus) was shown for 500 ms. Events in Stop trials was similar to those of Go trials, however, after go cue onset, a colored image (stop signal) replaced the go cue. The participants had to keep pressing the switch after seeing the stop signal. Failure to stop the response in Stop trials (switch release) was considered as an error and the error-signal was shown. Holding the switch pressed was considered as a correct response and all items disappeared on screen. (**b**) Participants performed the stop task in pre-tDCS and post-tDCS sessions. Participants put on a headphone and music (high-tempo, low-tempo, or no-music (background noise)) was played during the task performance (pre- and post-tDCS sessions) but not during brain stimulation.

Participants performed a computerized version of the stop task (Fig. 1a), that required inhibition of prepotent responses, before (pre-tDCS session) and after tDCS (post-tDCS session)^48^. In Go trials, a go-cue instructed a response toward a left or right target, however in Stop trials a stop signal (colored image with different emotional content: positive/negative/indifferent) appeared with a delay after the go cue (stop signal delay) and instructed the participants to inhibit their response. An adaptive procedure^2^ was used to keep the percentage of correct responses in Stop trials around 50% (Supplementary data). Different types of music, or none, was played while the subjects performed the stop task. Anodal or sham stimulation was delivered over DLPFC in a rest period between the pre- and post-tDCS sessions (Fig. 1b).

## Results

### The effects of practice and music on inhibition ability in Stop trials

Stop signal reaction time (SSRT) is a reliable index of inhibitory function and is calculated as a difference between mean response time (RT) and mean stop signal delay^2^. A lower SSRT indicates a better ability in inhibition of responses.

**Figure 2:**
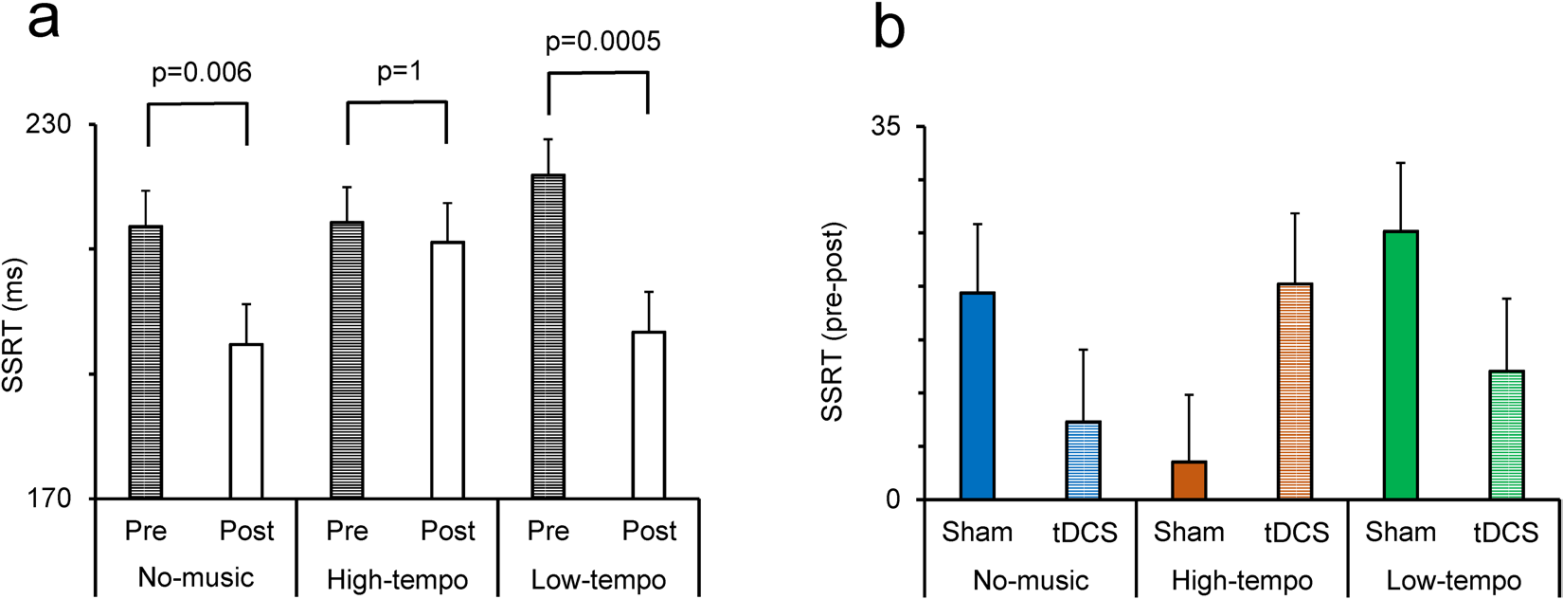
Modulation of Stop Signal Reaction Time (SSRT) by music and tDCS. (**a**) SSRT in pre and post sessions in sham condition for each music condition. Pairwise comparison of the differences between pre and post in each music condition showed a significant difference (two-tailed t test with Bonferroni adjustment for multiple comparison) between pre and post in no-music and low-tempo condition but not in the high-tempo condition. Practice-related changes in SSRT is seen in No-music and Low-tempo, but not in High-tempo, music conditions. (**b**) The difference in SSRT between pre and post sessions is shown in sham and tDCS conditions while participants were listening to different types of music. tDCS restored the practice-related changes in high-tempo music condition.

A two-way ANOVA [Pre-post (pre/post, within-subject factor) × Music-type (no-music/high-tempo/low-tempo, between-subject factor)] applied to the SSRT in sham condition showed that the main effect of Music-type was not significant however, the main effect of Pre-post was highly significant (F(1,70) = 17.57; p = 0.0001) (Partial Eta Squared = 0.20). This indicates that practice led to a decrease in SSRT (enhanced inhibition ability) in the post session (Fig. 2a). There was also a significant interaction between Pre-post and Music-type factors (F(2,70) = 3.06; p = 0.05) (Partial Eta Squared = 0.08) indicating that the rate of practice-related decrease in SSRT was dependent on the music type. Pairwise comparison of the differences between pre and post in each music condition showed a significant difference (two-tailed t test with Bonferroni adjustment for multiple comparison) between pre and post in no-music (p = 0.006) and low-tempo condition (p = 0.0006), but not in the high-tempo condition (p = 1). Figure 2a shows that the practice-related decrease in SSRT was abolished in high-tempo music condition.

### Interactive effects of music and tDCS in modulating inhibition ability in Stop trials

To examine whether tDCS and music interact in modulating the inhibition ability, a multifactor ANOVA [tDCS (sham/anode, within-subject factor) × Pre-post × Music-type × Emotion (positive/negative/indifferent, within-subject factor)] was applied to the SSRT. In ANOVA analyses, a significant main effect of tDCS indicates that the performance was different between sessions, which were separated by a week (washout period) but this does not necessarily point to a significant effect of tDCS on each behavioral measure. However, a significant three-way interaction between tDCS, Pre- vs post-tDCS and other factors indicates that the effects of the tDCS was dependent on the other factor. We hypothesized that if music and tDCS interact in modulating the behavioral measures, it would appear as a three-way interaction between tDCS, pre vs post and music factors. The main effect of tDCS, Emotion or Music-type was not significant however, the main effect of Pre-post (F(1,66) = 22.16; p = 0.0001) (Partial Eta Squared = 0.25) was highly significant (Fig. 2a,b). Figure 2b shows the difference in SSRT between the pre and post sessions (practice-related change). Importantly, there was a significant three-way interaction between tDCS, Pre-post and Music-type (F(2,66) = 4.39; p = 0.016) (Partial Eta Squared = 0.12) indicating that the tDCS significantly influenced SSRT but its modulatory effect was dependent on the background music (Fig. 2b). In sham condition, the practice-related change in SSRT was seen in no-music and low-tempo conditions but not in the high-tempo condition (Fig. 2a,b). However, these changes in SSRT were moderated after anodal stimulation in high-tempo music condition (Fig. 2b). These results suggest that the practice-related changes in inhibition ability were attenuated by high-tempo music, but restored by anodal stimulation. We further examined the underlying mechanisms of the interactive effects of practice, music and tDCS by measuring event-related changes in electrodermal activity (EDA) while participants performed the cognitive tasks.

### Arousal level was modulated by error and practice-related learning within stop task

**Figure 3:**
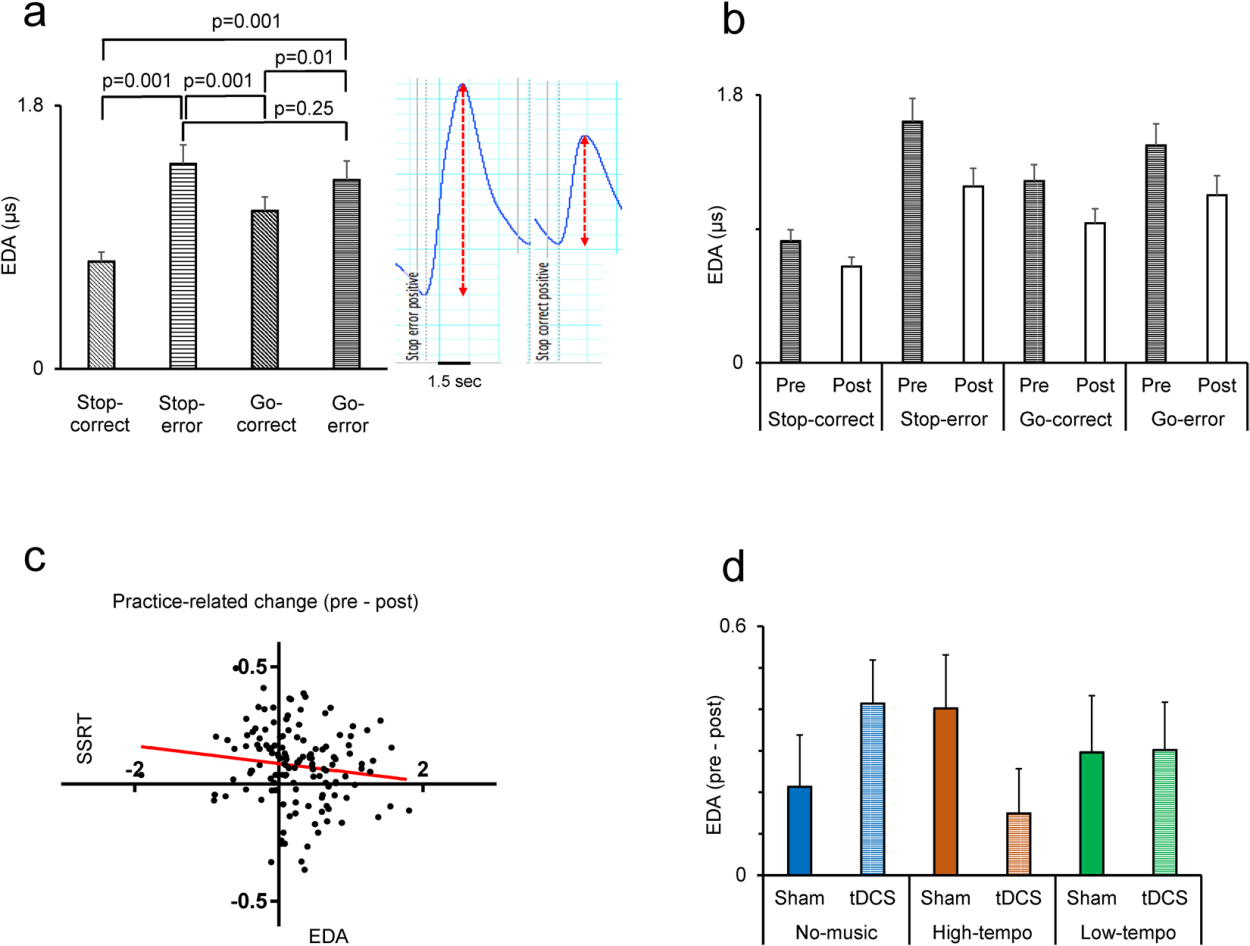
Modulation of electrodermal activity (EDA) by music and tDCS. (**a**) Event-related EDA in different trial-types. To estimate the EDA we calculated the maximum change in EDA in a 3-second time interval after a correct response in Go trials (Go-correct), after an error in Go trials (Go-error), after a successful response inhibition in Stop trials (Stop -correct) and after a failure in response inhibition in Stop trials (Stop-error). Right-side inset shows the representative phasic EDA waveform following feedback to error (left) or correct (right) responses. (**b**) EDA in pre and post sessions for each trial type. (**c**) Practice-related changes in SSRT and EDA were significantly correlated (two-tailed Spearman’s rho = −0.17, p = 0.047). Each dot indicate the values for each participant. The red line shows the fitted regression line. (**d**) The difference in EDA between pre and post sessions is shown in sham and tDCS conditions while participants were listening to different types of music.

Previous studies^47,49–51^ have shown that the task-related changes in electrodermal activity (skin conductance) might reflect the arousal/emotional state change during cognitive task performance. To estimate the event-related electrodermal activity we calculated the maximum change in EDA in a 3-second time interval^51^ after a correct response in Go trials (Go-correct), after an error in Go trials (Go-error), after a successful response inhibition in Stop trials (Stop -correct) and after a failure in response inhibition in Stop trials (Stop-error). The phasic EDA was aligned at the moments when the participants received feedback about the outcome of their response in Go and Stop trials (Fig. 3a inset). A two-way ANOVA [Response-type (Stop-error/Stop-correct/Go-correct/Go-error, within-subject factor) × Pre-post] applied to the mean EDA values in sham condition showed a highly significant main effect of Response-type (F(3,201) = 50.75; p = 0.0001) (Partial Eta Squared = 0.43). The largest EDA response was seen in Stop-error and Go-error trials (Fig. 3a). This indicates that stronger EDA responses were seen when the participants received feedback about their error commission suggesting that errors provoked a strong arousal/emotional response. The inset in Fig. 3a shows the EDA response following a failure (left) and success (right) in response inhibition. There was also a significant main effect of Pre-post (F(1,67) = 14.35; p = 0.0001) (Partial Eta Squared = 0.18) indicating that the EDA signal significantly decreased in the post session (Fig. 3b). This suggests that EDA response to feedback (behavioral outcome) significantly decreased after practice. There was also a significant interaction between Response-type and Pre-post (F(1,67) = 5.23; p = 0.002) (Partial Eta Squared = 0.07) indicating that the rate of practice-related decline in EDA response differed between the trials. The largest practice-related decline was seen in Stop-error trials (Fig. 3b).

Whereas the practice-related decline in SSRT shows an improvement in inhibition ability, a decline in EDA response indicates that the arousal/emotional reaction to decision outcome (error or correct) decreased after practice. We hypothesized that a practice-related decrease in EDA response to feedback would impair the learning from feedback and consequently lead to a decrease in practice-related improvement in inhibition ability. Therefore, we assumed that a negative correlation must exist between the practice-related changes in SSRT (decline in SSRT) and EDA responses (decline in EDA response): A larger practice-related decline in EDA would lead to a smaller practice-related decline in the SSRT. There was a significant negative correlation between the EDA and SSRT changes (two-tailed Spearman’s rho = −0.17, p = 0.047) indicating that a larger practice-related decline in EDA response was accompanied by a smaller decline in SSRT (smaller improvement in inhibition ability) (Fig. 3c).

### The interactive effects of tDCS and music on arousal/emotional state in Stop trials

To gain insight into the underlying mechanisms of the interaction of tDCS and music in modulating SSRT we examined the interactive effects of tDCS and music in modulating the EDA responses in Stop trials. We applied a multifactor ANOVA [tDCS × Pre-post × Music-type × Emotion] to the EDA response in Stop-error trials. There was a significant main effect of Pre-post (F(1,64) = 21.57; p = 0.0001) (Partial Eta Squared = 0.25) but no significant main effect of Emotion or Music. More importantly, there was a significant three-way interaction between tDCS, Pre-post and Music-type (F(2,64) = 3.49; p = 0.037) (Partial Eta Squared = 0.10) indicating that the tDCS significantly influenced EDA response but its modulatory effect was dependent on the background music (Fig. 3d). Figure 3d shows the difference in EDA between the pre and post session and therefore a larger value indicates a larger practice-related decline in EDA response. A close examination of Figs 2b and 3d indicates a parallel but opposite pattern of practice-related changes between the SSRT and EDA responses. High-tempo music attenuated improvement in inhibition ability and simultaneously increased the decline in EDA response, so that in post sessions the participants’ arousal/emotional response to feedback was very low. Such a decline in responsiveness to decision outcome might reflect/underlie the attenuated learning induced by high-tempo music. Intriguingly, the anodal stimulation moderated the decline in EDA response and concomitantly restored the practice-related decrease in SSRT in high-tempo music condition. This suggests that anodal stimulation prevented the decline in arousal/emotional response to errors and therefore reinstated the ability to learn and improve inhibition ability in the presence of high-tempo music. Importantly, such interactive effects between music and tDCS in modulating the EDA was absent in a period before participants received feedback to their decision and was not seen in Stop-correct trials (Supplementary data). These indicate that music and tDCS specifically modulated the EDA (arousal) response to the decision outcome and particularly after a failure in response inhibition.

### Interactive effects of music and tDCS in modulating response time in Go trials

**Figure 4:**
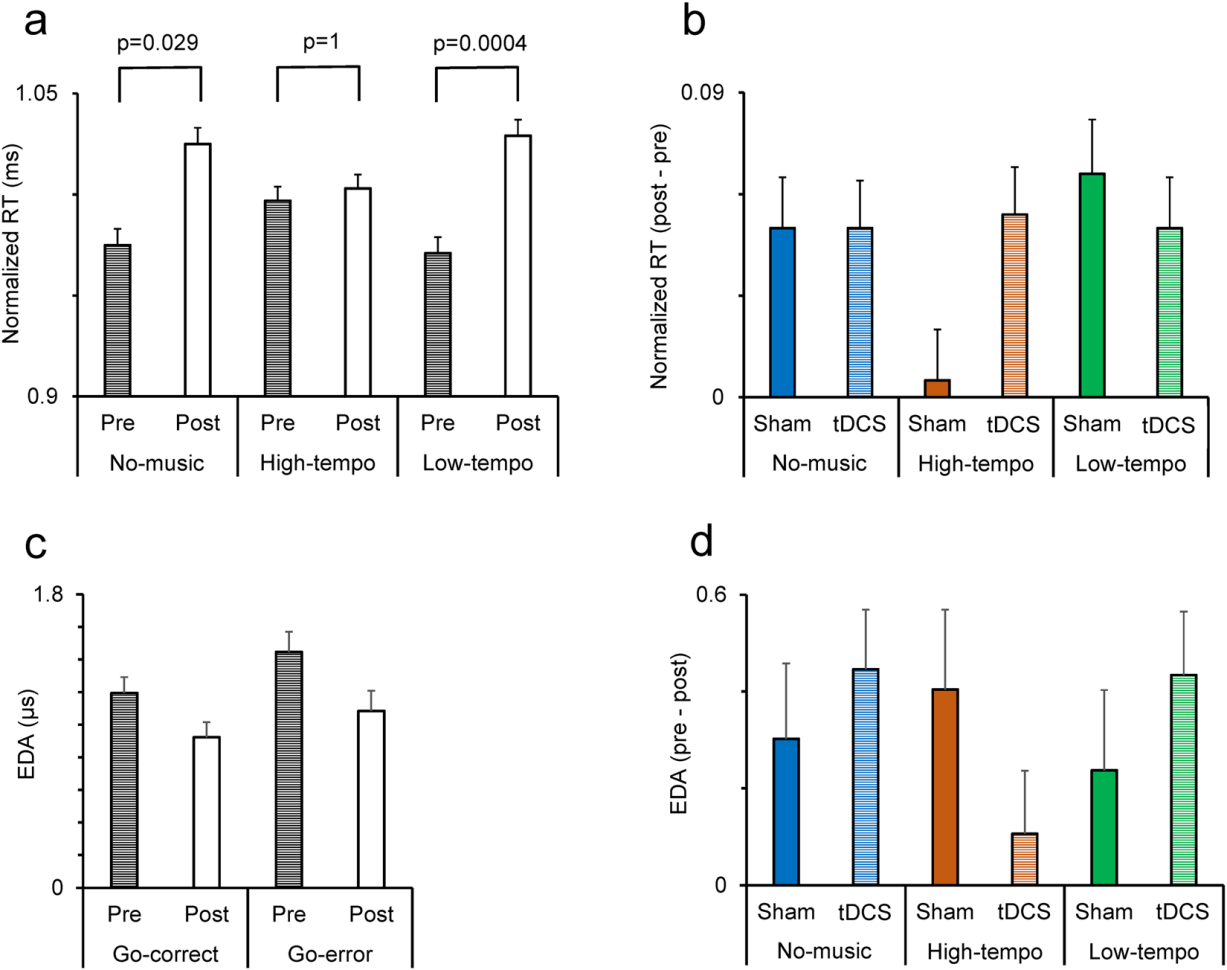
Modulation of response time (RT) and EDA by music and tDCS. (**a**) RT in pre and post sessions in sham condition for each music condition. (**b**) The difference in RT between post and pre sessions (post - pre) is shown in sham and tDCS conditions while participants were listening to different types of music. (**c**) RT in pre and post sessions in Go-correct and Go-error trials. (**d**) The difference in EDA between pre and post sessions (pre - post) is shown in sham and tDCS conditions while participants were listening to different types of music.

Previous studies^2,31^ have shown that response time (RT) in Go trials depends on the demand for response inhibition in the preceding trials. We compared those Go trials that were preceded by another Go trial (CgCg; C = correct, g = Go trial) with those Go trials that were preceded by a failed inhibition in Stop trial (EsCg; E = error, s = Stop trial) and found that response time (RT) in Go trials was significantly modulated by the demand for inhibition in the preceding Stop trials (Supplementary material). Therefore, the effects of music and tDCS was separately analyzed in Go trials that were preceded by Stop or Go trials. We examined the modulatory effects of tDCS and Music by applying a multi-factor ANOVA [Trial-type (CsCg/EsCg, within-subject factor) × tDCS × Prepost × Emotion (positive/negative/indifferent stimuli shown in the preceding Stop trial, within-subject factor) × Music-type] to the normalized RT in Go trials. The ANOVA showed that there was no significant main effects of tDCS, Emotion or Music. However, the main effect of Trial-type (F(1,68) = 34.06; p = 0.0001) (Partial Eta Squared = 0.33) and Prepost (F(1,68) = 46.65; p = 0.0001) (Partial Eta Squared = 0.41) was highly significant showing that RT was slower after a successful response inhibition. Importantly, a significant three-way interaction was found between the tDCS, Prepost and Music (F(2,65) = 3.87; p = 0.026) (Partial Eta Squared = 0.10) indicating that the tDCS significantly influenced RT in Go trials but its modulatory effect was dependent on the music type (Fig. 4b). Figure 4b shows the difference in RT between the pre-tDCS and post-tDCS testing (post-tDCS – pre-tDCS). In sham condition, there was a practice-related increase in RT in no-music and low-tempo conditions however, this practice-related change was abolished when participants were listening to high-tempo music. The practice-related change in RT became larger after anodal stimulation in high-tempo, but not in no-music or low-tempo conditions. These indicate that the practice-related changes in RT was attenuated by high-tempo music, but restored by anodal stimulation.

### The interactive effects of tDCS and music on arousal/emotional state in Go trials

We examined whether EDA was modulated by tDCS and music in Go trials. A multi-factor ANOVA [Response-type (Go-error/Go-correct) × tDCS × Prepost × Music-type] was applied to the EDA response in Go trials. The main effect of Response-type (F(1,64) = 28.12; p = 0.0001) (Partial Eta Squared = 0.31) was significant showing that the EDA response was larger in Go-error trials. There was also a significant main effect of Prepost (F(1,64) = 18.56; p = 0.0001) (Partial Eta Squared = 0.23) indicating that EDA significantly decreased in post session suggesting a practice-related decrease in EDA response to feedback in Go trials (Fig. 4c). There was also a significant interaction between Response-type and Prepost factors (F(1,64) = 5.15; p = 0.027) (Partial Eta Squared = 0.07) indicating that the rate of practice-related decrease in EDA response was significantly higher in Go-error trials (Fig. 4c).

More importantly, there was a significant three-way interaction between tDCS, Prepost and Music (F(2,64) = 3.54; p = 0.035) (Partial Eta Squared = 0.10) indicating that the tDCS significantly influenced EDA response but its modulatory effect was dependent on the background music condition (Fig. 4d). A close examination of Fig. 4b and d indicates a parallel but opposite pattern of changes between EDA and the RT. The practice-related change in RT was small in high-tempo music condition however, it was restored by tDCS; correspondingly, the practice-related change in EDA was large in high-tempo music condition but decreased after anodal tDCS. This suggests that high-tempo music attenuated the practice-related slowing of response and concomitantly increased the decline in responsiveness to feedback in a way that in post sessions the participants’ arousal/emotional response to feedback was very low. Such a decline in responsiveness to errors might reflect the attenuated learning by high-tempo music. Intriguingly, the anodal stimulation restored the practice-related slowing of RT in high-tempo music condition and concomitantly decreased the decline in responsiveness to the feedback in Go trials. This suggests that anodal stimulation prevented the decline in arousal/emotional response to feedback and therefore reinstated the strategic slowing of RT in the presence of high-tempo music. We also examined whether there was a correlation in practice-related changes between the RT and the EDA response in Go trials. There was a negative correlation between the RT and EDA values, however it did not reach significance (two-tailed Spearman’s rho = −0.08, p = 0.38).

## Discussion

### Interactive effects of music and tDCS on performance and arousal level

Our findings indicate that practice induced remarkable behavioral adjustments that appeared as a decline in SSRT and an increase in RT in Go trials (Figs 2a and 4a). Such practice-related changes might reflect a strategic adjustment in behavior that aimed at optimizing performance in the stop task. The practice-related decline in SSRT indicates that participants learned to improve their inhibition ability in Stop trials. The practice-related slowing in Go trials might also reflect a learning process that led to a more cautious performance in Go trials in order to better detect and respond to the stop signal. These practice-related changes were influenced by listening to different music types. Whereas strong practice-related changes in SSRT and RT occurred in no-music and low-tempo music, listening to the high-tempo music blocked such learning for both SSRT and RT. The tDCS influenced both the SSRT and RT, but in both cases the tDCS effects were dependent on the music type (Figs 2b and 4b). We also found parallel practice-related changes in EDA which were significantly influenced by listening to music. The largest decline in EDA response to the decision outcome was seen in high-tempo music suggesting that high-tempo music led to a decline in arousal response, and possibly in efficient assessment of the decision outcome and consequently decreased the learning process. These findings suggest that tDCS restored emotional response to the decision outcome and therefore reinstated the learning and improvement in inhibition ability in the presence of high-tempo music.

### Music, but not visual stimuli, influenced cognitive functions in stop task

In our study, two contextual factors, the emotional content of the stop cue and the background music, could potentially modulate the emotional state of the participants. Both factors, from different modalities, were not relevant to the cognitive task performance. The emotional content of images used as the stop cue did not affect the performance in Stop trials or in the Go trials that followed the Stop trials as evidenced in no significant main effect of Emotion or its interaction with music or tDCS in the ANOVA tests. The high-tempo or low-tempo music was played as background music and therefore participants could simply ignore the background music and concentrate on the ongoing task. However, in contrast to images, high-tempo music appeared as a salient cognitive factor that significantly attenuated learning to inhibit the inappropriate response. No-music condition included low-intensity background noise without any particular pattern or musical elements. The pattern of practice-related changes in inhibition ability and EDA responses was similar between the no-music and low-tempo conditions. However, the behavioral effects and the concomitant EDA changes were different between the high-tempo condition and the other two music conditions.

### Subjective feelings/emotions evoked by the high- and low-tempo music

The songs used as background music were first selected as a collection of pop music in a previous study^31^ and then sorted (using a software by two different investigators) into high- and low-tempo songs for the current study. Therefore, there was no bias in selecting and assigning the songs to high- or low-tempo categories. The effects of high-tempo music cannot be simply attributed to the higher sound intensity or lyrics because low-tempo music had the same intensity and also contained lyrics. The high- and low-tempo songs might also differ in other aspects of music and evoke different subjective feelings/emotions and therefore we conducted a ‘Control study’ to systematically examine whether other aspects of background music such as ‘Preference’, ‘Familiarity’, ‘Lyrics’ or ‘Novelty’ differed between the high-tempo and low-tempo music conditions and led to the significant interactions with the tDCS in modulating arousal and executive functions. Findings of this control test clearly indicate that subjective perception of preference, familiarity, lyrics and novelty did not differ between the high- and low-tempo music conditions (see Fig. S3 and supplementary data). This confirms that the significant interactions between music conditions and tDCS in modulating the arousal level and performance in stop task were unrelated to these aspects of the background music.

### Music influenced task-related changes in arousal/emotional state

Previous studies have reported contradicting findings regarding the modulation of participants EDA response while they perform cognitive tasks^52–55^ or listen to different types of music^16^. Increases and also decreases in EDA, depending on the subjective feeling of pleasure or arousal, have been reported when participants listen to background music^52,55^. In our study, measuring event-related EDA response to decision outcome (errors) helped to gain insight into the possible mechanisms through which music and tDCS modulate the inhibition ability. ANOVA analyses applied to the SSRT or RT or EDA showed that the main effect of Music was not significant but the interaction between Music and Prepost and the interaction between Music, Prepost and tDCS factors were significant. This indicates that music did not necessarily change the SSRT or RT or EDA, instead it mainly influenced the practice-related learning process for the SSRT, RT and EDA. This suggests that music did not directly affect the motivation or executive control processes. However, high-tempo music significantly enhanced the practice-related decline in EDA response to the decision outcome. Our findings suggest that high-tempo music altered the arousal response to the decision outcome and consequently attenuated the practice-related learning. This assumption conforms to Somatic marker hypothesis^53^, which has proposed that alterations in autonomic and arousal responses can potentially influence learning and decision processes.

### The link between arousal/emotion and executive functions: a converging point for the interactive effects of music and prefrontal cortex stimulation

We found an intriguing interaction of tDCS and high-tempo music. Anodal stimulation of prefrontal cortex restored practice-related improvement in inhibition ability which had been abolished by the high-tempo music. A close examination of the effects of tDCS (Fig. 1b) indicates that tDCS did not necessarily enhance the practice-related improvement in inhibition ability in all music conditions. The practice-related change was actually decreased by anodal stimulation when it was applied in no-music and low-tempo music conditions, however tDCS augmented such practice-related changes in the high-tempo music condition. This suggests that tDCS and high-tempo music show interactive effects that could not be seen in the absence of music or with low-tempo condition. A similar pattern of interactive effects of tDCS and high-tempo music appeared in modulating the EDA response to the decision outcome (Fig. 3d). tDCS did not necessarily change the practice-related decline in EDA response in all music conditions. The tDCS was remarkably effective in blocking the effects of high-tempo music and restoring the arousal response to the decision outcome. A recent study has examined tDCS over right DLPFC on music performance of novice and expert performers^56^ and found that overall, tDCS had no main effect but showed interaction with the level of expertise so that tDCS benefitted novice musicians but hindered the experts. These findings suggest that the modulatory effects of tDCS might depend on the level of practice and experience.

We propose that the effects of high-tempo music on learning and implementation of inhibitory functions, at least in part, was mediated through alterations in the assessment of decision outcome and in the related learning process. The interactive effects of tDCS in restoring the effects of high-tempo music was also mediated, at least in part, through restoring the arousal response to the decision outcome. Our findings do not necessarily establish a causal link between the changes in arousal response to the decision outcome and the inhibition ability, and neither show which one originated first to drive the other one. However, the parallel and concomitant changes in the arousal response to the decision outcome and in inhibitory function by high-tempo music and by anodal stimulation suggest that changes in arousal response and inhibition ability were closely associated and emerged from interaction of emotional state and executive functions^57,58^. We found a significant correlation in the practice-related alterations between arousal response and inhibition ability (Fig. 3c), which conforms well to the predictions of the somatic marker hypothesis^53,59^. The high-tempo music led to a significant decrease in arousal response to the decision outcome and concomitantly blocked learning and implementation of inhibition ability. The pattern of tDCS effects indicate that tDCS moderated the blocking effects of high-tempo music on the arousal response and therefore restored the learning and improvement of inhibition ability.

### The neural mechanisms mediating the interactive effects of music and brain stimulation

Two scenarios might explain the neural substrate and mechanisms underlying the interactive effects of practice, music and tDCS. The first scenario, closely aligned with the somatic marker hypothesis^53,59^, assumes that alterations in emotional state influence the learning and decision process and prefrontal cortical circuits, and particularly OFC, mediate the relationship. We found a significant correlation in the practice-related alterations between arousal response and inhibition ability (Fig. 3c), which conforms well to the predictions of the somatic marker hypothesis. The high-tempo music led to a significant decrease in arousal response to the decision outcome and concomitantly blocked learning and implementation of inhibition ability. Absence of such arousal response might have impaired the learning process and improvement in inhibition ability as predicted by somatic marker hypothesis^53^. In this scenario music might have influenced different neural structures such as amygdala and ACC that are potentially involved in arousal and autonomic responses to the task-related events and context^31,60,61^ without necessarily affecting neural networks that support executive control processes. A recent study in monkeys^62^ reports that pupil size, as an index of arousal level, predicts the upcoming behavioral performance and that ACC cell activity correlated well with pupil size suggesting that ACC is involved in adjusting the autonomic aspects of arousal in relation to cognitive task performance. This scenario needs to explain how anodal stimulation over DLPFC counteracted the music effects on both the arousal response and inhibition ability. The DLPFC, ACC and OFC are parts of a distributed executive control network, which is closely involved in assessment of errors and in guiding executive control of behavior^1,61,62^. DLPFC and ACC are also involved in outcome-based learning for action selection and in adjusting arousal and autonomic responses during cognitive task performance^1,31,61–70^. Anodal tDCS might elevate the sensitivity of executive control network to the influence of arousal signal and therefore counteract the effects of music, however this assumption does not fully explain our findings because we did not find a tDCS-induced increase in arousal response in no-music or in low-tempo conditions. Instead the pattern of tDCS effects indicate that tDCS moderated the blocking effects of high-tempo music on the arousal response and therefore restored the learning and improvement of inhibition ability. An alternative scenario assumes that high-tempo music directly influenced the executive network (including DLPFC and ACC) and by engaging parts of cognitive resources led to impaired learning and inhibition ability^71^. tDCS might also directly influence this network and therefore both music and tDCS interact on the same neural substrate to influence the inhibition ability. In this scenario the concomitant changes in the arousal response reflected the autonomic aspects of the processes that are involved in assessment of the decision outcome without necessarily influencing the decision or learning per se. We propose that our findings conform better to the first scenario.

The neural mechanisms underlying the effects of high-tempo music on response inhibition and arousal level in the stop task are still unclear. Our control study indicated that high- and low-tempo music were perceived and categorized as happy and sad songs, respectively by the majority of participants. Music might influence overall mood and emotional regulation^6,9^ and indirectly affect the learning and improvement in inhibition ability. Alternatively, music might directly influence the executive network (including DLPFC and ACC) and by engaging parts of cognitive resources lead to impaired performance^71^. tDCS might also directly influence this network and therefore both music and tDCS interact on the same neural substrate to influence the inhibition ability. In this scenario the concomitant changes in the arousal response reflected the autonomic aspects of the processes that are involved in assessment of the decision outcome without necessarily influencing the decision or learning per se (see supplementary discussion).

Deficits in executive functions might underlie learning disabilities and compulsive behavior such as addiction^3,41^. Our findings, for the first time, show that both music and tDCS interactively alter arousal-emotional response to the decision outcome and consequently influence executive functions. Further studies need to evaluate how combination of music and tDCS might be used as a safe and non-invasive method in rehabilitation of neuropsychiatric and compulsive disorders.

## Methods

All experiments were approved by Human Research Ethics Committee of Monash University and all methods were performed in accordance with the relevant guidelines and regulations. All participants gave written informed consent for their participation in the study. Participants with current or history of neurological or neuropsychiatric disorders were not included in this study. Participants who reported sleep deprivations, a history of seizures, pregnancy, excessive caffeine use, previous brain imaging or the use of psychoactive medicines that affect cognitive abilities were excluded. Participants performed the stop task while in the presence or absence of different types of background music in pre- and post-tDCS sessions. The tDCS of DLPFC was applied in a randomized counter-balanced and crossover (repeated-measure) design during a rest period between the pre- and post-tDCS sessions while there was no background music.

### Apparatus

A computerized version of stop task was used^48^ in which participants were seated in front of a touchscreen and a switch. All visual stimuli were shown on the touchscreen. The experiment and data acquisition were controlled by CORTEX program (National Institute of Mental Health) at millisecond (ms) resolution. Participants were instructed to perform the Stop task (Fig. 1a) as fast and accurately as possible. Participants responded by pressing and releasing the switch and touching the items on the touchscreen (Microtouch). Participants’ performed the task individually in a separate test room, however their behaviour and hand movements were monitored by a video camera from a control room. The participants wore a wireless headphone during the cognitive task performance. To ensure consistency, participants read an explanatory statement about the test procedure and received a structured verbal briefing in the first session of testing.

### Cognitive task and experimental procedure

Participants: 73 right-handed English-speaking university students (37 females and 36 males; aged 18–32 years) were recruited for this study. They were divided into three music conditions [no-music (12 female, 12 males), high-tempo (13 females, 12 males) and low-tempo (12 females, 12 males)].

#### Stop task

In each trial, after the onset of a start cue participants had to press on a switch (Fig. 1a)^48^. Switch press led to the onset of a fixation point (for 350 ms), and then two target items (small white circles) appeared on the right and left side of the screen (for 300 ms). If participants kept the switch pressed, a go cue was shown at the centre of the touchscreen as a signal to initiate the motor response. A vertical or horizontal white bar was presented as the go cue, which instructed the left target or right target selection, respectively. After the onset of go cue, participants had to respond as fast as possible by releasing the switch and touching the correct target item on the touchscreen within a limited (900 ms from the onset of the go cue) time window. After a correct selection, feedback was provided (the target item turned off (for 200 ms) and then on (for 200 ms) two times). Feedback was given (all items disappeared and an error signal was presented for 500 ms) after an erroneous response (Fig. 1a).

In Stop trials, within trial events and contingencies were the same as in Go trials, however a stop signal (an image) was shown with a delay after the go cue. Stop signal instructed participants to inhibit their response and keep holding the switch pressed. We also classified the Stop trials based on the emotional content of the image shown as the stop signal to positive, negative or indifferent (neutral). Images with different emotional content were shown randomly with equal proportion. Each image was shown only once in each testing session. One female and one male experimenter rated the emotional content of each image before its inclusion in the stop signal set. The emotional content of the stop signal was not relevant to the task performance and the participants were instructed to inhibit their response if any image appeared following the go cue. The interval between the stop signal onset and go cue onset (stop signal delay, SSD) was adjusted depending on the success of participants in inhibiting their response in the preceding Stop trial. The SSD in the first stop trial was 15 ms (the stop signal was presented 15 ms after the onset of the go cue). If participants were successful in inhibiting their response, the SSD would increase by 40 ms in the next Stop trial each time. However, if participants failed in response inhibition, the SSD would remain at 15 ms or decrease by 40 ms if the SSD had previously increased. This adaptive procedure (performance dependent SSD adjustment) was independently applied in the negative, indifferent and positive Stop trials. The adaptive procedure adjusts the difficulty of inhibition in stop trials so that an accuracy level of around 50% is expected. The adaptive procedure in this stop task was effective in bringing the performance close to this expected value. After completion of several practice trials, all participants successfully completed totally 500 correct trials in each daily session (250 correct trials in each pre- and post-tDCS testing). In 30% of trials, the Stop signal was shown which means that in each testing session there were about 75 correct Stop trials with left or right response requirement. Therefore, about 300 erroneous and 300 correct responses in Stop trials (75 × 2 (pre- and post-tDCS) ×2 (daily sessions)) were collected from each participant and used for data analyses. Stop and Go trials were intermixed and presented randomly. All items disappeared and an error signal was presented if participants failed in response inhibition. For calculation of the percentage of correct responses in Stop trials, the first 4 Stop trials in each testing session were excluded.

Trials in which a response to the left or right side was required were run randomly and in the same proportion. If participants did not touch the items on the screen within the time window all the items disappeared and an error signal was presented. Touching the wrong target which did not match the instructed direction by the go cue was classified as an error. Early release of the switch before the onset of the go cue was also classified as an error. All items disappeared after an error and a visual feedback (error-signal) was presented for 500 ms. The interval from the onset of the go cue to the release of switch was considered as the response time (RT). The switch release, but not the screen touch, was considered as the response because it could not be influenced by the preferential use of a particular finger or hand^48^. Pressing and releasing the switch was monitored at millisecond resolution. A practice block in which only Go trials were presented was run at the beginning of testing session. If participants attained 90% accuracy within 20 consecutive trials in the practice block, trials of the main block were run. Data from these main blocks were used for analyses.

### Music conditions

Participants were classified into three music groups and listened to music through wireless headphones during the cognitive task performance. Among various music features that affect the emotional state, the tempo is better quantifiable and less subjective (Supplementary discussion) and therefore different music pieces were classified into two separate categories of high-tempo and low-tempo music. In high-tempo and low-tempo music condition, contemporary pop music (with lyrics) at 120–140 and 80–100 beats per minute (BPM) was played, respectively. In no-music condition the participants put on the headphones and could hear the muffled noise coming from the computer, nearby rooms and the click of the switch but no background music was played. The selection criteria for the songs were absence of any offensive statement related to religion, sex or ethnicity in the lyrics. The same set of songs was played, in a random order, for each participant. We did not sort the songs according to the participants’ preference to keep the consistency in the music played for each participant. We set the volume for all the subjects at 70 decibels, but the subjects were allowed to adjust it if they found it too low or too high.

### tDCS procedure

TDCS was delivered over the DLPFC of participants who performed the stop task before (pre-tDCS) and after tDCS (post-tDCS) (Fig. 1b). Participants rested on a chair and no music was played during tDCS application. Anodal tDCS was applied through a positive 2.5 cm × 4 cm sponge electrode (saline-soaked) on the DLPFC in the left (dominant) hemisphere, and a larger negative 4 cm × 6 cm electrode over the supraorbital area in the right hemisphere^44,48,72^. Handedness was confirmed by Edinburgh Handedness Questionnaire and all participants were right-handed. Electrode positioning followed the international 10–20 system for locating the DLPFC based on skull landmarks (F3). Direct current (1.5 mA for 10 minutes), with 15 seconds of linear fade in and fade out, was delivered by a tDCS device (Intelect® Advanced Therapy System, Chattanooga, USA). Participants sat silently and every 3 minutes had to write their subjective experience of tDCS side effects on a standard sheet while the tDCS was applied.

Participants performed the stop task before and after tDCS. The tDCS was delivered while participants were not performing the stop task. The post-tDCS stop task performance started 5 minutes after the end of the stimulation to ensure that cognitive task performance started after a fixed period following the tDCS in all participants. Electrodes and attachments were detached and participants rested during this 5 minutes. Two separate stimulation sessions (sham and anodal tDCS) were considered for each participant (repeated measure). These two tDCS sessions were separated by a washout period (1 week). We counterbalanced the order of tDCS sessions (sham/anode) across participants to control for any tDCS order or practice effects. Stimulation type (anode or sham) was not revealed to participants. In sham condition, a conventional approach^44,48,72^ was implemented for blinding by applying transient current (the first 15 seconds fade in, 30 seconds constant 1.5 mA and 15 seconds fade out). The polarity of stimulation in sham condition was randomly changed across participants. To assess the effectiveness of the blinding procedure, at the end of testing session, participants were asked to guess whether they received tDCS in that session. In average, 60.3% of participants correctly guessed the stimulation type (sham or anode) in the first and the second sessions. To monitor side/adverse effects of tDCS, all participants completed a questionnaire to indicate the severity of side effects such as burning sensations, headache, tingling, itching and pain by reporting numeric analogue scales (e.g., 0 = no tingling to 10 = worst tingling imaginable)^44,48,72^. All participants completed the stimulation sessions without reporting any significant side or adverse effects (headache, burning sensation or pain). Most participants indicated a transient tingling and itching under the smaller electrode. In a few participants, slight transient redness was seen beneath the electrodes.

### Electrodermal activity recording procedure

Electrodermal activity (EDA) was measured by an electrodermal recording unit (ML116 GSR Amp- ADInstruments). The EDA amplifier (sampling rate of 75 kHz) was attached to PowerLab (26 T) recording units for monitoring and storage of data. During cognitive task performance skin conductance was continuously recorded by two metal electrodes attached to the palmar surface of the index and ring fingers of the non-dominant hand. The participants were instructed to avoid moving their non-dominant hand and keep it on a pad over the desk. Conductivity measurement was displayed in Standard International conductance units (microsiemens). EDA was monitored in real time and event codes were automatically included through the behavioral control software (CORTEX) for assessing the event-related phasic changes in the EDA signal. Amplitude of phasic activity was measured as the difference between the maximum and minimum value of the EDA waveform within a 3 second window following each event^51^ (Fig. 3a inset). In 5 participants the EDA response could not be reliably recorded in all sessions (due to factors such as motion artefact, cold hand, very low or very high levels of sweating) and therefore their data were excluded from the analyses (two observers blind to the analyses results assessed the EDA records in each participant and decided about inclusion or exclusion of data).

### The procedure for examining the subjective feelings/emotions induced by high- and low-tempo music

In order to assess whether other aspects of songs (music) such as ‘Preference’, ‘Familiarity’, ‘Lyrics’ or ‘Novelty’ differed between the high-tempo and low-tempo music, we conducted an additional control test.

#### Participants and assessment procedure

This test was conducted in 14 participants (6 males and 8 females) who were in the same age range of the participants of the main tDCS study. These participants were recruited based on advertisement and paid for their contribution. They listened to the same 14 high-tempo and 14 low-tempo songs, which were used in the tDCS study, and ranked them based on Preference (scale of 1–5), Familiarity (scale of 1–5), interest in Lyrics (no/neutral/yes), perceived Novelty (old/neutral/new) and Happiness/Sadness (sad/neutral/happy) associated with each song. The participants had to listen to the entire song and complete a form. The high- and low-tempo songs were intermingled and randomly played for the participants. Participants were not aware of the classification of songs based on the tempo or the order of played songs. During rating of the songs no other task performance was required.

This assessment of subjective perception of each song could not be done in the main brain stimulation study because in the tDCS study the songs (high- or low-tempo songs) were played as background music while participants were performing the cognitive tasks. Therefore, participants could not assess and record the subjective impression for each individual song while they were focused in performing the challenging cognitive tasks. This assessment of songs could not be done at the end of the cognitive task performance because participants listened to about 20 songs during the task performance and the assessment of subjective impression of each song based on memory could not be reliable. The results of this control test is presented in the supplementary material.

### Data analyses and statistical approaches

In Go trials, the response time (RT) was measured as the interval between the onset of the go cue and the switch release and then averaged separately for each condition. In all the Analysis of Variance tests (ANOVA), we used raw data (percentage of correct responses or response time) without any transformation or removing outliers. However, to facilitate comparison of the different sessions and groups in figures, RT values were normalized in each condition by dividing it by the mean RT in all the conditions in that session. The effects of practice, emotional images and tDCS on various behavioral measures were assessed by repeated-measure ANOVA. Mauchly’s Test was used to examine sphericity and Greenhouse-Geisser correction was applied when necessary. Two-tailed t test with Bonferroni adjustment for multiple comparisons were used for all pairwise comparisons. Music was played in different groups and therefore was included in the ANOVA as a between-subject factor. Images with different emotional content (positive/negative/neutral) were shown as the stop signal in Stop trials. Although, the emotional content of these images were not related to the actual task performance, we assumed that music might modulate the significance of the emotional content of these images and consequently influence the performance in Stop trials. Therefore, Emotion was included in the ANOVA as a within-subject factor. tDCS was included as a within-subject factor because we examined the tDCS (sham and anode) effects in each participant.

#### Correlation analyses

We examined whether there was a correlation between the practice-related behavioral changes and the EDA response (SSRT vs EDA in Stop trials or RT vs EDA in Go trials). For each participant, the difference between pre and post sessions (pre – post) was normalized by dividing it by their mean and then used for correlation analyses.

In ANOVA analysis, Partial Eta Squared indicates the proportion of the variance explained by the effect and was reported for each significant effect. For all statistical results p ≤ 0.05 was considered as significant.

## Electronic supplementary material

Supplementary material
