# Supplementary material for: Interactive effects of music and prefrontal cortex stimulation in modulating response inhibition
Source: Sci Rep. 2017 Dec 22;7:18096. doi: 10.1038/s41598-017-18119-x (PMC5741740; doi:10.1038/s41598-017-18119-x)
Supplement: Supplementary file 1 — Supplementary material [file 41598_2017_18119_MOESM1_ESM.pdf]

Supplementary material

Title: Interactive effects of music and prefrontal cortex stimulation in modulating response inhibition

Authors: Farshad A. Mansouri, Nicola Acevedo, Rosin Illipparampil, Daniel J. Fehring, Paul B. Fitzgerald, Shapour Jaberzadeh.

Supplementary data

Percentage of correct responses in Stop trials:

The adaptive procedure in Stop trials was intended to control the percentage of correct responses around 50%. A three-way ANOVA [Pre-post x Music-type x Emotion], applied to the percentage of correct responses in sham condition, showed that the main effect of Music or Emotion or their interaction with Pre-post factor was not significant. However, there was a significant main effect of Pre-post ($F(1,70) = 4.82$; $p = 0.03$) (Partial Eta Squared = 0.06) indicating that there was a small practice-related decline in the percentage of correct responses from the pre (mean + SE, 53.62 ± 0.47) to the post (52.18 ± 0.46) session, yet the adaptive procedure kept the performance around 50% (Fig. S1a). No interaction between these factors was seen.

Percentage of correct responses in Go trials:

A three-way ANOVA [tDCS (sham/anode) x Pre-post x Music-type], applied to the percentage of correct responses in Go trials, showed that the main effect of Music or Pre-post or Music factors or their interactions were not significant (Fig. S1b).

Response time in Go trials depends on the demand for response inhibition in the preceding trials

Previous studies[2, 31, 48] have reported that following a stop trial there is a slowing of the response in the subsequent Go trial. We compared those Go trials that were preceded by another Go trial (CgCg; C = correct, g = Go trial) with those Go trial that were preceded by a failed inhibition in Stop trial (EsCg; E = error, s = Stop trial). A two-way ANOVA [Trial-type (CgCg/EsCg) x Pre-post] applied to the RT in Go trials in sham condition showed that there was a significant main effect of Trial-type ($F(1,72) = 103.65$; $p = 0.0001$) (Partial Eta Squared = 0.59) indicating a significant response slowing following Stop-error trials (Fig. S2a). The main effect of Pre-post was also significant ($F(1,72) = 7.92$; $p = 0.006$) (Partial Eta Squared = 0.10) indicating a significant practice-related increase in RT (Fig. 3b). There was no significant interaction between Trial-type and Pre-post factors. Another ANOVA comparing CgCg trials with a Go trial that was preceded by a successful inhibition in Stop trials (CsCg) showed that there was a significant main effect of Trial-type ($F(1,72) = 145.96$; $p = 0.0001$) (Partial Eta Squared = 0.67) indicating a significant response slowing following Stop-correct trials (Fig. S2b). The main effect of Pre-post was also significant ($F(1,72) = 14.30$; $p = 0.0001$) (Partial Eta Squared = 0.17) indicating a significant practice-related

increase in RT. There was no significant interaction between Trial-type and Pre-post factors. These analyses indicated that RT in Go trials was significantly modulated by the demand for inhibition in the preceding Stop trials and therefore the effects of music and tDCS was separately analyzed in Go trials that were preceded by Stop or Go trials.

Interactive effects of music and tDCS in modulating response time in Go trials

We first examined whether music type influenced the RT in sham condition. A three-way ANOVA [Trial-type (CsCg/EsCg) x Pre-post x Music-type] showed that the main effect of Music was not significant however, the main effect of Trial-type was significant ($F(1,70) = 27.02$; $p = 0.0001$) (Partial Eta Squared = 0.28) indicating that RT was slower after a successful inhibition of response in Stop trials. The main effect of Pre-post was also highly significant ($F(1,70) = 19.19$; $p = 0.0001$) (Partial Eta Squared = 0.22) indicating that practice led to an increase in RT in the post session. Importantly, there was a significant interaction between Pre-post and Music-type factors ($F(2,70) = 3.51$; $p = 0.035$) (Partial Eta Squared = 0.09). This indicates that RT significantly increased by practice however, the rate of this increase was dependent on the music type (Fig. 4a). Pairwise comparison of the differences between pre and post sessions in each music condition showed a significant (two-tailed t test with Bonferroni adjustment for multiple comparison) difference between pre and post in no-music ($p = 0.029$) and low-tempo condition ($p = 0.0004$), but not in the high-tempo condition ($p = 1$). This indicates that the practice-related increase in RT was abolished in high-tempo music condition (Fig. 4a). No significant interaction was seen between Trial-type, Pre-post and Music indicating that the practice-related effect of music did not differ between trial-types.

We also applied a multi-factor ANOVA [Trial-type (CsCg/EsCg, within-subject factor) x tDCS x Pre-post x Emotion (positive/negative/indifferent stimuli shown in the preceding Stop trial) x Music-type] to the normalized RT in Go trials that were preceded by correct Go trials (CgCg) and found that while there was a significant main effect of Pre-post ($F(1,70) = 21.78$; $p = 0.0001$) (Partial Eta Squared = 0.24), there was no significant three-way interaction between tDCS, Pre-post and music.

Arousal level was modulated by error commission and practice

A two-way ANOVA [Response-type (Stop-error/Stop-correct/Go-correct/Go-error, within-subject factor) x Pre-post] applied to the mean EDA values in sham condition showed a highly significant main effect of Response-type ($F(3,201) = 50.75$; $p = 0.0001$) (Partial Eta Squared = 0.43). Pairwise comparison of the differences between response-types showed a significant (Bonferroni adjustment for multiple comparison) difference in EDA between Stop-error and Stop-correct ($p = 0.001$), between Stop-error and Go-correct ($p = 0.001$), between Stop-correct and Go-correct ($p = 0.001$), between Stop-correct and Go-error ($p = 0.001$), between Go-correct and Go-error ($p = 0.01$), but not between Stop-error and Go-error ($p = 0.25$) trials.

Interactive effects of music and tDCS on arousal level was not seen in Stop-correct trials

We applied the four-way ANOVA [tDCS x Pre-post x Music-type x Emotion] to the EDA response in Stop-correct trials. Although there was a significant main effect of Pre-post ($F(1,65) = 19.11$; $p = 0.0001$) (Partial Eta Squared = 0.23), no significant three-way interaction between tDCS, Pre-post and Music was seen ($F(2,65) = 1.27$; $p = 0.29$). This suggests that the interactive effects of tDCS and music modulates EDA only in Stop-error trials (after a failure in response inhibition).

Electrodermal activity in a period before the arrival of feedback to the decision outcome was not modulated by Music or tDCS

For the main analyses, phasic EDA response was measured within a 3 second window following feedback to the correct or erroneous responses (after-feedback EDA) and we found that music and tDCS interactively modulated such after-feedback EDA. In separate analyses we also calculated the EDA response in a 3 seconds period before feedback to correct or erroneous response (from -3 sec to the feedback time) in Go and Stop trials (before-feedback EDA). We hypothesized that the interaction of tDCS and Music in modulating the SSRT or response time would be seen in the EDA response following feedback (after decision outcome) but not before that. An ANOVA [tDCS (sham/anode) x Pre-post x Music-type x Emotion] was applied to the before-feedback EDA signal in Stop-error trials. There was a significant main effect of Pre-post ($F(1,64) = 12.39$; $p = 0.001$) (Partial Eta Squared = 0.16) indicating that before-feedback EDA was decreased by practice. However, there was no significant three-way interaction between tDCS, Pre-post and Music-type ($F(2,64) = 0.55$; $p = 0.58$) (Partial Eta Squared = 0.017) indicating that in contrast to the after-feedback EDA, the before-feedback EDA was not modulated by music and tDCS.

Another ANOVA [Response-type (Go-error/Go-correct) x tDCS x Pre-post x Music-type] was applied to the before-feedback EDA in Go trials. There was a significant main effect of Pre-post ($F(1,64) = 15.20$; $p = 0.0001$) (Partial Eta Squared = 0.19) indicating that before-feedback EDA significantly decreased by practice in Go trials. However, there was no significant three-way interaction between tDCS, Pre-post and Music-type ($F(2,64) = 0.69$; $p = 0.51$) (Partial Eta Squared = 0.021) indicating that in contrast to the after-feedback EDA, the before-feedback EDA in Go trials was not modulated by music and tDCS.

Results of a control study to examine subjective feelings/emotions induced by high- and low-tempo music

All 14 participants completed the ranking for all the 28 songs. For each music factor such as familiarity we conducted a separate Chi-Square test of independence to examine whether there was an association between the music tempo (High/Low) and other subjective aspects of music such as preference or familiarity. A contingency table was formed for each factor. The null hypothesis to be tested is that the music tempo is independent of the other factor (such as Familiarity); that is the music tempo is not associated with the subjective perception of familiarity.

We first examined whether there was an association between the music tempo and Preference factor. No significant association was found between the music tempo and participants' Preference ($X^2_{0.05, 4} = 2.15$; $p = 0.71$) (Figure S3a). There was no significant association between the music tempo and Familiarity ($X^2_{0.05, 4} = 1.16$; $p = 0.88$) (Figure S3b). There was no significant association between the music tempo and Lyrics ($X^2_{0.05, 2} = 4.27$; $p = 0.12$) (Figure S3c). There was no significant association between the music tempo and Novelty ($X^2_{0.05, 2} = 4.72$; $p = 0.09$) (Figure S3d).

Previous studies[73-79] have shown that music tempo is associated with mood changes and induces different levels of happiness/sadness and therefore we asked participants to rate the songs based on their perception of happiness/sadness associated with each song (sad/neutral/happy). As predicted, participants associated high-tempo and low-tempo music with happy and sad categories, respectively, which appeared as a highly significant association between the music tempo and perceived happiness/sadness of the songs ($X^2_{0.05, 2} = 29.47$; $p = 0.0001$) (Figure S3e). This indicates that participants paid attention to categorizing

each song and our analytical approach was sensitive enough to dissociate subjective perception of music.

In our main tDCS study participants performed the cognitive tasks in different music conditions (high-tempo music, low-tempo music or background noise) and we found a significant interaction between music condition and tDCS effect in modulating the cognitive task performance and arousal response (EDA). Findings from our control test clearly indicate that subjective perception of ‘preference’, ‘familiarity’, ‘lyrics’ and ‘novelty’ did not differ between the high- and low-tempo music conditions. This confirms that the observed interaction of tDCS and music condition was not arising from these non-specific aspects of music. We also confirmed that our control test was sensitive enough to detect the well-known effect of music tempo in inducing positive and negative mood states[73-79].
We conclude that the significant interactions of music conditions (high-tempo/low-tempo/low-noise) with tDCS in modulating the arousal level and performance in executive control tasks were unrelated to the familiarity, preference, novelty or lyrics of the background music.

Supplementary discussion

The neural mechanisms mediating the effects of music on inhibition ability

The impact of high-tempo music on inhibition ability might be attributed to the effects of music on separate cognitive processes with dissociable neural substrate: (i) High-tempo music might change the emotional state and mood[7, 9] and consequently influence motivation to better perform the cognitive tasks. Therefore, the performance in post session might decline and mask the practice-related improvement in inhibition ability. (ii) Music might directly engage attentional resources and influence the executive functions through modulating activity in DLPFC, OFC and ACC[32-35] and therefore prevent practice-related plastic changes that normally lead to learning and improvement in inhibition ability. (iii) Music might influence neural networks that are involved in the reward processing and prediction[45] and induce emotional alterations through inducing reward prediction violations[52]. Therefore, while participants are involved in performing an ongoing task and prediction of its behavioral outcome, background high-tempo music might engage these processes and influence the assessment of behavioral outcome (correct or error) and attenuate the learning process. (iv) Somatic marker hypothesis[53] proposes that the cognitive demand for making a

choice and its associated uncertainty are accompanied by alterations in autonomic and arousal responses, which influence learning and decision processes. Patients with OFC lesion show impairment in the arousal response that is normally expressed in response to risky and uncertain decisions[47]. It is proposed that such arousal responses facilitate evaluation of decision outcome and strategic adjustments that optimize performance in volatile and uncertain conditions[47]. In our study, high-tempo music might influence the neural network involved in regulation of autonomic and arousal responses[13] or influence ACC and medial prefrontal areas that are involved in regulating arousal responses during cognitive task performance[54] and therefore change the arousal response to the decision outcome (errors) that normally guide the learning process. Such a decline in arousal response to decision outcome might hinder the practice-related improvement in inhibition ability.

Interaction of tDCS with learning processes

A recent study suggest that combining tDCS and singing[80-81] improves speech recovery in stroke patients. Previous studies have shown that cathodal stimulation over auditory cortical areas impaired participants' performance in pitch memory tasks[81-84]. However, the effects of anodal stimulation have been mixed showing improvement[83, 84] or impairment[85] in pitch memory tasks. A recent study examined the effects of anodal and cathodal tDCS on pitch learning and showed that anodal, but not cathodal, stimulation impaired learning across daily sessions. Other studies have also shown that tDCS might influence learning process in other domains such as motor skill[84, 85], language and verbal working memory[86, 87]. tDCS might have positive or negative effects on feedback-based learning processes[88] depending on stimulation specifications and contextual factors. The modulatory effects of tDCS over DLPFC on musical creativity has been recently reported where tDCS improved it in less-experienced musicians but hindered it in experienced musicians[89]. It has been shown that tDCS over cerebellum modulated the music effect on line bisection ability in healthy participants suggesting that tDCS changes the neural network state and its susceptibility to the music effects[90]. These studies suggest that tDCS induces changes in neural networks and consequently alter their susceptibility for learning-related plastic changes[44, 88].

Supplementary figure legend

Supplementary figure 1. **Percentage of correct responses in Stop and Go trials**

(a) Percentage of correct responses in Stop trials in pre and post sessions while the participants listened to different types of music. (b) Percentage of correct responses in Go trials in pre and post sessions.

Supplementary figure 2. **Response showing after Stop trials**

(a) Normalized response time (RT) is shown in Go trials that were preceded by another Go trial (CgCg; C = correct, g = Go trial) and those Go trials that were preceded by a failed inhibition in Stop trial (EsCg; E = error, s = Stop trial). (b) Normalized response time (RT) is shown in CgCg trials and those Go trials that were preceded by a successful inhibition in Stop trial (CsCg).

Supplementary figure 3. **Subjective feeling/emotions induced by high- and low-tempo music.**

Subjective impression provoked by high- and low-tempo songs in terms of ‘Preference’, ‘Familiarity’, ‘Lyrics’, ‘Novelty’ and ‘Happiness/Sadness’. Vertical axis shows the incidence rate of each condition.

Supplementary references

73. Hunter, P. G., Schellenberg, E. G. & Schimmack, U. Feelings and perceptions of happiness and sadness induced by music: Similarities, Differences, and Mixed Emotions. Psychology of Aesthetics, Creativity, and the Arts. 4, 47–56 (2010).

74. Fernández-Sotos A., Fernández-Caballero A., Latorre J. M. Influence of tempo and rhythmic unit in musical emotion regulation. Front Comput Neurosci. 10:80 (2016).

75. Gabrielsson, A., & Juslin, P. Emotional expression in music. In R. Davidson, K. Scherer, & H. Goldsmith (Eds). The handbook of affective sciences (pp. 503–534). New York: Oxford University Press. (2003).

76. Mote, J. The effects of tempo and familiarity on children's affective interpretation of music. Emotion 11, 618–22 (2011).

77. Gagnon, L. & Peretz, I. Mode and tempo relative contributions to "happy-sad" judgments in equitone mequitone. Cognition & Emotion 17, 25–40 (2003).

78. Hevner, K. The affective value of pitch and tempo in music. American Journal of Psychology 49, 621–630 (1937).

79. Webster, G. D. & Weir, C. G. Emotional responses to music: Interactive effects of mode, texture, and tempo. Motivation and Emotion 29, 19–39 (2005).

80. Vines, B.W., Norton, A.C. & Schlaug, G. Non-invasive brain stimulation enhances the effects of melodic intonation therapy. *Frontiers in Psychology* 2, 230 (2011).

81. Mathys, C., Loui, P., Xin, Z. & Schlaug, G. Non invasive brain stimulation applied to heschl's gyrus modulates pitch discrimination. *Frontiers in Psychology* 193, 1-7 (2010).

82. Schaal, N.K., Williamson, V.J. & Banissy, M.J. Anodal transcranial direct current stimulation over the supramarginal gyrus facilitates pitch memory. *The European Journal of Neuroscience* 38, 3513-3518 (2013).

83. Tang, M.F. & Hammond, G.R. Anodal transcranial direct current stimulation over auditory cortex degrades frequency discrimination by affecting temporal, but not place, coding. *The European Journal of Neuroscience* 38, 2802-2822 (2013).

84. Antal, A., et al. Facilitation of visuo-motor learning by transcranial direct current stimulation of the motor and extrastriate visual areas in humans. *The European Journal of Neuroscience* 19, 2888-2892 (2004).

85. Reis, J., et al. Noninvasive cortical stimulation enhances motor skill acquisition over multiple days through an effect on consolidation. Proceedings of the *National Academy of Sciences of the United States of America* 106, 1590-1595 (2009).

86. Flöel, A., Rösser, N., Michka, O., Knecht, S. & Breitenstein, C. Noninvasive brain stimulation improves language learning. *J. Cogn. Neurosci*. 20, 1415-1422 (2008).

87. Reinhart R.M.G. & Woodman G.F. Causal control of medial–frontal cortex governs electrophysiological and behavioral indices of performance monitoring and learning. *Journal of Neuroscience* 34, 4214-4227 (2014).

88. Filmer, H.L., Mattingley, J.B., & Dux, P.E. Improved multitasking following prefrontal tDCS. *Cortex* 49, 2845-2852 (2013).

89. Rosen, D.S., Erickson, B., Kim, Y.E., Mirman, D., Hamilton, R.H., Kounios, J. Anodal tDCS to right dorsolateral prefrontal cortex facilitates performance for novice jazz improvisers but hinders experts. *Frontiers Human Neuroscience* 16, 10-579 (2016).

90. Picazio, S., Granata, C., Caltagirone, C., Petrosini, L., Oliveri, M. Shaping pseudoneglect with transcranial cerebellar direct current stimulation and music listening. *Frontiers Human Neuroscience* 9, 158 (2015).

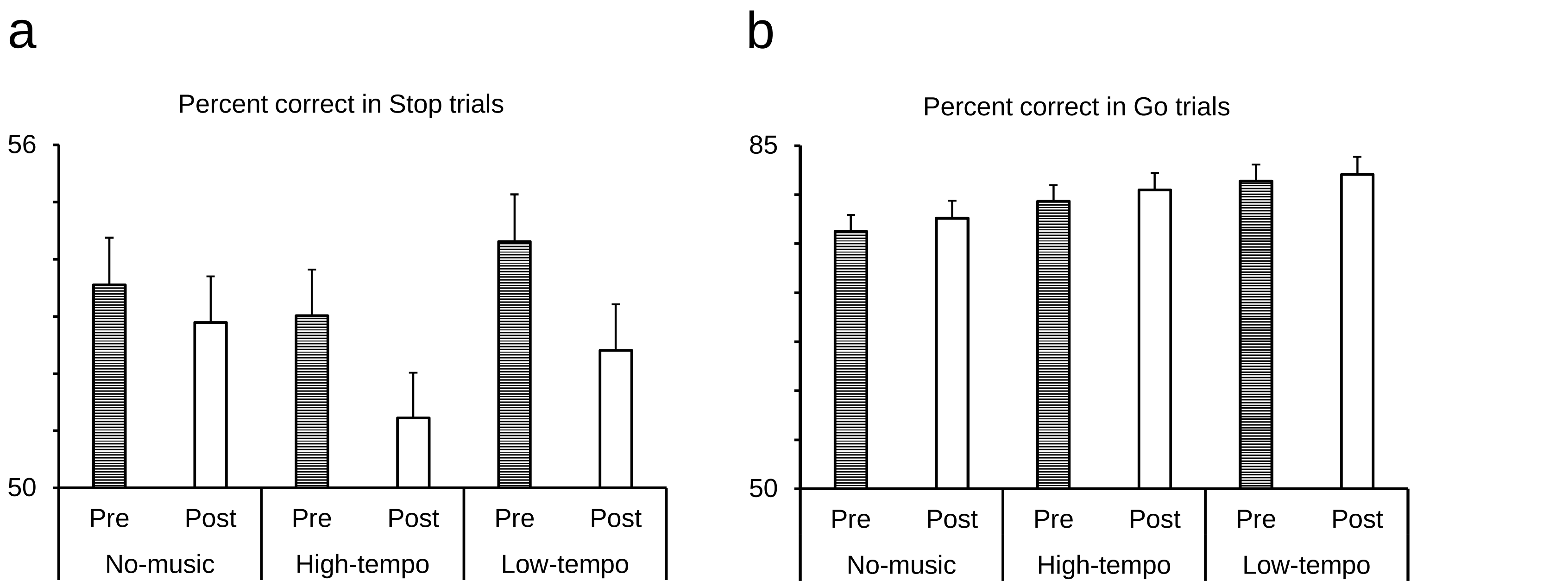

Supplementary figure 1. Percentage of correct responses in Stop and Go trials

(a) Percentage of correct responses in Stop trials in pre and post sessions while the participants listened to different types of music. (b) Percentage of correct responses in Go trials in pre and post sessions.

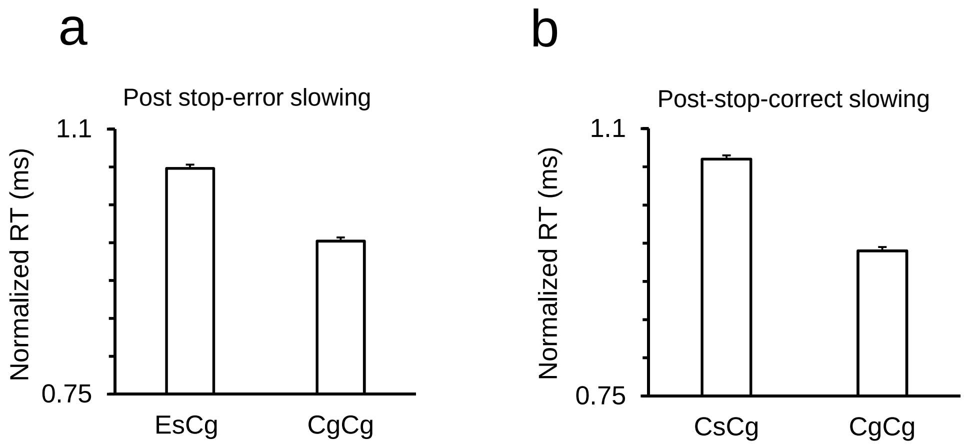

Supplementary figure 2. **Response showing after Stop trials**

(a) Normalized response time (RT) is shown in Go trials that were preceded by another Go trial (CgCg; C = correct, g = Go trial) and those Go trials that were preceded by a failed inhibition in Stop trial (EsCg; E = error, s = Stop trial). (b) Normalized response time (RT) is shown in CgCg trials and those Go trials that were preceded by a successful inhibition in Stop trial (CsCg).

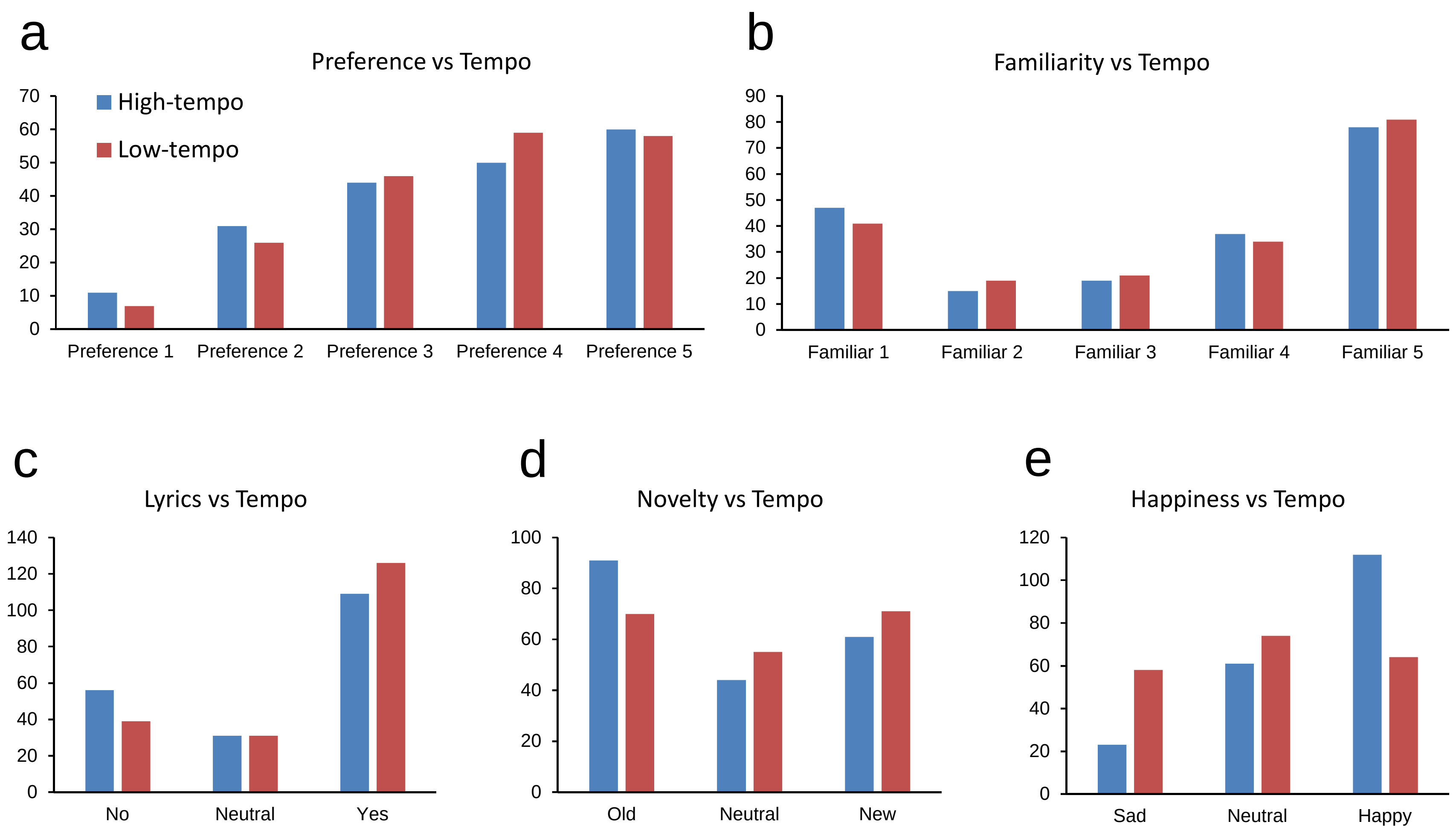

Supplementary figure 3. Subjective feeling/emotions induced by high- and low-tempo music.
Subjective impression provoked by high- and low-tempo songs in terms of ‘Preference’, ‘Familiarity’, ‘Lyrics’, ‘Novelty’ and ‘Happiness/Sadness’. Vertical axis shows the incidence rate of each condition.
